# MTV, an ssDNA Protecting Complex Essential for Transposon-Based Telomere Maintenance in *Drosophila*

**DOI:** 10.1371/journal.pgen.1006435

**Published:** 2016-11-11

**Authors:** Yi Zhang, Liang Zhang, Xiaona Tang, Shilpa R. Bhardwaj, Jingyun Ji, Yikang S. Rong

**Affiliations:** 1 State Key Laboratory of Bio-control, Institute of Entomology, School of Life Sciences, Sun Yat-sen University, Guangzhou, China; 2 Laboratory of Biochemistry and Molecular Biology, National Cancer Institute, National Institutes of Health, Bethesda, Maryland, United States of America; Geisel School of Medicine at Dartmouth, UNITED STATES

## Abstract

Multiple complexes protect telomeres. In telomerase-maintained organisms, Shelterin related complexes occupy the duplex region while the CST and Tpp1-Pot1 complexes bind the single stranded overhang of telomeres. Drosophila uses a transposon-based mechanism for end protection. We showed that the HOAP-HipHop complex occupies the duplex region. Whether an ssDNA-binding complex exists is not known. Here we discover a novel protein, Tea, that is specifically enriched at telomeres to prevent telomere fusion. We also identify a complex consisting of Tea and two known capping proteins, Ver and Moi. The Moi-Tea-Ver (MTV) complex purified *in vitro* binds and protects ssDNA in a sequence-independent manner. Tea recruits Ver and Moi to telomeres, and point mutations disrupting MTV interaction *in vitro* result in telomere uncapping, consistent with these proteins functioning as a complex *in vivo*. MTV thus shares functional similarities with CST or TPP1-POT1 in protecting ssDNA, highlighting a conserved feature in end protecting mechanisms.

## Author Summary

Chromosome ends are protected by the telomere structure maintained by the telomerase enzyme in most organisms. The fruit fly Drosophila has fascinated the field as the only major model organism that relies solely on a telomerase-independent mechanism for end protection. The fly model is arguably the best system to reveal the most basic features of the telomere. Here we characterize the MTV complex in flies and suggest that MTV fulfills similar function as the ssDNA-binding complexes in other organisms. This is striking considering that MTV subunits display highly accelerated rates of protein evolution. Our findings will be of interest to scientists interested in the molecular mechanisms of telomere protection and evolution biologists interested in how telomere and telomeric functions evolve.

## Introduction

The ends of linear chromosomes are protected by the structure called Telomere. Although the necessity of end protection is universal, how this capping function is fulfilled can differ in different organisms. Above all, the underlying DNA sequence at telomeres can vary greatly. In most cases studied, telomeric DNA consists of short repeats synthesized by the telomerase enzyme [reviewed in 1]. In these organisms, the repeats also serve as binding sites for protein complexes that protect and regulate telomere functions [reviewed in 2, 3]. If telomere shortening is not prevented beyond a critical extent, capping is lost leading to cellular senescence or genome instability in case of continuing proliferation. Telomere capping proteins in general show high degree of divergence at the primary sequence level even among organisms with very similar telomeric repeats (e.g. mammals *vs*. higher plants). Furthermore, DNA sequences identical to the telomeric repeats are present internally. Therefore, primary DNA sequence is not sufficient to confer capping function; other features at telomeres, perhaps chromatin structures, are needed for normal end protection.

The most extreme case of DNA sequence divergence at telomeres can be found in many insect species including the model *Drosophila melanogaster*. Drosophila lacks telomerase and their chromosome ends are populated with telomere-specific retro-transposons [reviewed in 4]. We and others have shown that telomere protection in Drosophila can be entirely sequence-independent under laboratory conditions [5 and references therein]. Chromosome ends without transposons can be maintained indefinitely, and *de novo* attachment of telomeric transposons can occur at these ends. These results suggest that Drosophila telomere capping might represent the most primitive mode of end protection, the study of which is likely to reveal fundamental features of chromosome ends that distinguish them from ends of broken DNA.

Towards this goal, we identified a Drosophila capping complex that contains HipHop [6] and HOAP [7; 8]. This complex is specifically enriched at all telomeres, and its loss leads to extensive end-to-end fusion. We further showed using ChIP that HOAP-HipHop occupies a large domain of the double stranded region of telomere [6]. These features of HOAP-HipHop resemble those of the Shelterin-related complexes in other eukaryotes, suggesting that although sharing no homology at the sequence level they might be functionally homologous.

Raffa et al. [9] showed that the Drosophila capping protein Ver shares structural similarities with Stn1, which is a subunit of the conserved CST (Cdc13/Ctc1-Stn1-Ten1) complex that protects telomeres [reviewed in 10]. Remarkably, we showed that Ver is important for the recruitment of retro-transposon RNPs to telomeres, a process functionally similar to the targeting of telomerase to chromosome ends [11], highlighting a potential significance for the homology between Ver and Stn1.

In most organisms studied, the telomere ends as single stranded DNA with a 3’ overhang. This feature is believed to be essential not only for end protection but also for telomere elongation by telomerase [reviewed in 12]. The CST complex from diverse organisms maintains ssDNA homeostasis at telomeres. In addition, the mammalian TPP1-POT1 complex contributes to the protection of telomeric ssDNA and telomerase recruitment [13–15]. Whether Drosophila telomeres end in an overhang remains unknown. However, the identification of Ver as a potential Stn1 homolog suggests the intriguing possibility that they do and that a functionally similar CST or TPP1-POT1 complex exists in Drosophila.

In this study we identify a new capping protein Tea that prevents telomere fusion. We show that Tea forms a complex with the previously identified Ver and Moi proteins. A purified Moi-Tea-Ver (MTV) complex exhibits sequence-independent affinity for ssDNA. Upon binding to MTV, ssDNA is protected from exonuclease activities *in vitro*. Our results thus uncover additional conservations between telomerase-based and transposon-based mechanisms of end protection.

## Results

### TEA is a new capping protein in Drosophila

**Fig 1:**
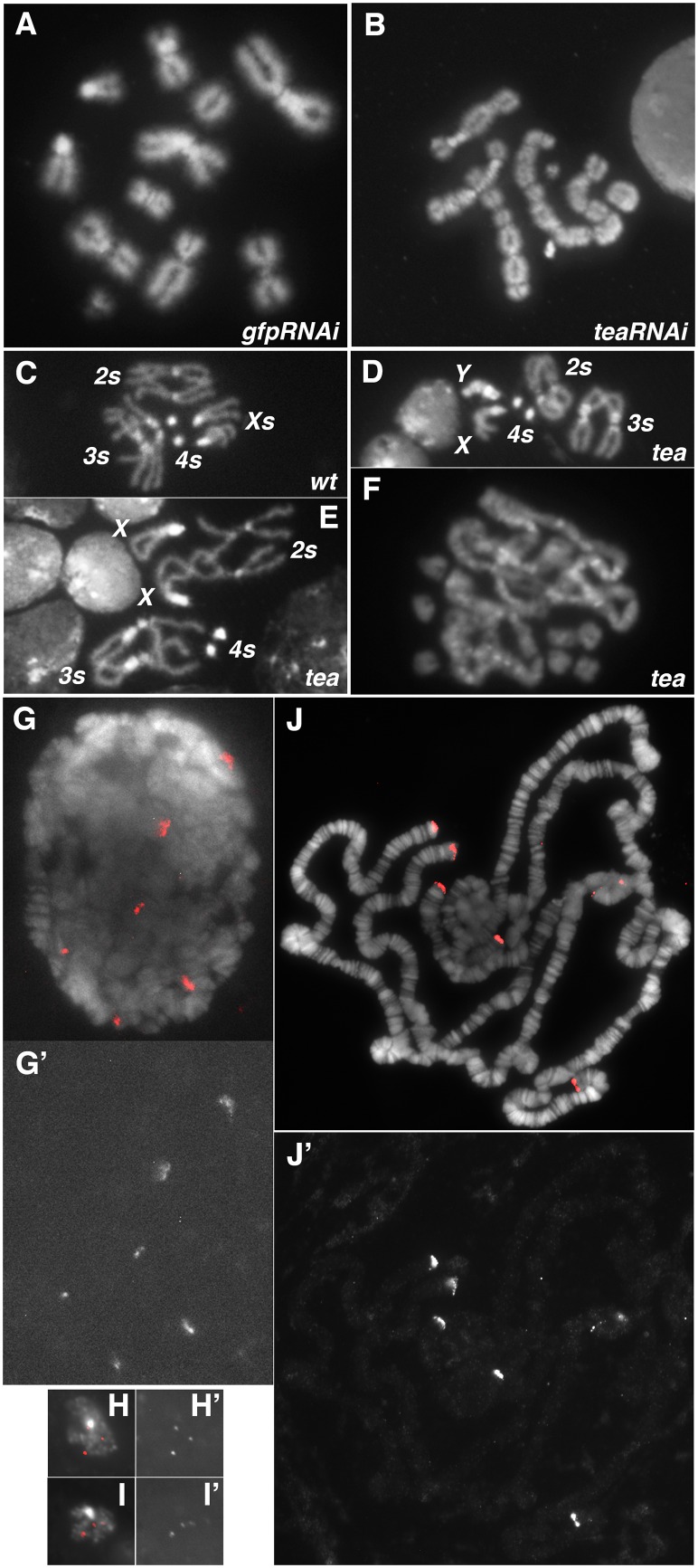
Tea localizes to telomeres for their protection. **A, B**. Knocking down *tea* expression leads to telomere fusion in cultured cells. **A**: a control cell treated with RNAi agents against *egfp* showing no telomere fusion of mitotic chromosomes. **B**: a cell treated with RNAi agents against *tea* showing fusion. **C**: a female wildtype neuroblast showing normal chromosomes. **D-F**: mitotic chromosomes from *tea* mutants showing various degrees of end-to-end fusion. **F** shows a *tea*-mutant nucleus with essentially all chromosomes participating in end fusion. **G, G’,H, H’,I,I’**. Tea forms foci that are possibly telomeric. In **G’, H’** and **I’**, white signals depict live GFP fluorescence from either a polytene cell (**G’**) or a diploid neuroblast (**H’** and **I’**) expressing EGFP-Tea. In **G**, **H** and **I**, GFP signals in red are merged with DAPI signals for DNA. **J, J’**. Tea is specifically enriched at telomeres of polytene chromosomes. In **J’**, white signals depict anti-GFP signals from animals expressing EGFP-Tea. In **J**, anti-GFP signals in red are merged with DNA signals. Genotype of mutants: **D**, *tea*^*1755/2-1*^; **E** and **F**, *tea*^*1755*^*/Df* (*Df* is a chromosomal deficiency of the *tea* locus.).

We have taken a biochemical approach to identify factors essential for telomere protection in Drosophila. Through our efforts of isolating HOAP-interacting proteins, we identified HipHop as a new capping component [6]. In the same biochemical purification, we identified peptides encoded by the *CG30007* locus. RNAi knock down of *CG30007* expression in Drosophila S2 cells led to end fusion (Fig 1B) suggesting that *CG30007* encodes a new capping protein. We named the locus *t**elomere* *e**nds* *a**ssociated* or *tea* for short. We discovered that the *l(2)1755* mutation recovered previously [16] might be a mutant allele of *tea* since animals heterozygous for *l(2)1755* and a chromosomal deficiency of the *tea* region (*l(2)1755/Df*) die as larvae and have cells suffering end fusion (Fig 1D, 1E and 1F). We examined 90 mutant nuclei and observed an average telomere fusion frequency of 6.7 with all nuclei showing at least one fusion. This extent of telomere dysfunction is one of the strongest amongst telomere uncapping mutants [17]. When we introduced into *l(2)1755/Df* flies a 10kb genomic fragment of the wild type *tea* locus via *P* element mediated transformation, we were able to rescue viability and eliminate end fusion (S1 Fig in Supplemental Materials). We therefore named *l(2)1755* as *tea*^*1755*^. Genomic sequencing of *tea*^*1755*^ identified a *CAG* to *TAG* change leading to a premature stop codon (S1 Fig), as well as other changes that might be SNPs. We recovered two additional *tea* alleles (Supplemental Materials) and all combinations of *tea* alleles, including those with the chromosomal deficiency, cause lethality accompanied by telomere fusion, phenotypes that in each case are rescued by the wildtype transgene.

Tea homologous proteins have not been identified outside of Drosophila, a feature consistent with a faster rate of protein evolution, which is shared by many Drosophila factors important for telomere maintenance such as HOAP, HipHop, Moi and Ver [reviewed in 18]. Although sequence alignments of Drosophila Tea homologs revealed blocks of conserved amino acid residues, analyses with available structural prediction programs did not yield identifiable domains in Tea.

### Tea localizes to telomeres and regulates other components of the capping machinery

To determine the cellular localization of Tea, we created a *tea* locus that encodes a Tea protein with its N-terminus fused to EGFP using the SIRT gene targeting method that we previously developed [19; Supplemental Materials]. In live interphase cells from larval diploid tissues, GFP-Tea forms foci reminiscent of interphase localization of other telomeric proteins such as HOAP and HipHop (Fig 1H and 1I; ref [20; 21]). In live larval polytene cells from the salivary gland, GFP-Tea forms 5–6 distinct strips in the nucleus likely representing telomeres on euchromatic chromosome arms (Fig 1G). This is again similar to GFP-HOAP localization. We then stained polytene chromosomes with an anti-GFP antibody and unambiguously localized Tea to telomeres (Fig 1J). Taken collectively, these results suggest that the Tea protein is specifically localized at telomeres, a property similar to several other proteins involved in telomere capping: HOAP, HipHop, Moi, and Ver.

Using mutants and *egfp*-tagged endogenous loci, we investigated the relationship between Tea and these other capping proteins with regards to their localization to telomeres. To monitor HOAP and HipHop, we used previously characterized antibodies and a previously made *gfp-cav* locus, which encodes HOAP [6; 20; 21]. For Ver, we constructed a *ver* locus with an N-terminally tagged *egfp* gene using SIRT (*gfp-ver*, Supplemental Materials). Live fluorescent signals from GFP-Tea and GFP-Ver were much weaker than that from GFP-HOAP indicating that neither Tea nor Ver is abundant. The localization of these proteins was monitored in interphase diploid cells and polytene cells using live fluorescence from GFP.

**Fig 2:**
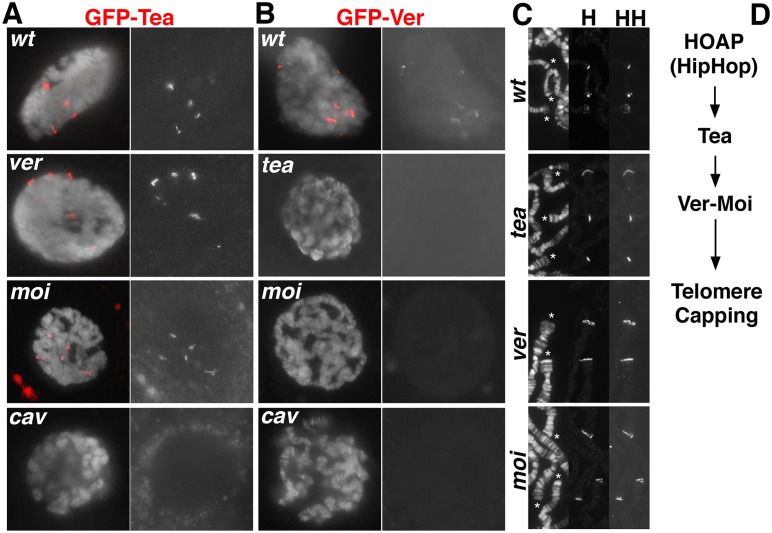
Genetic control of capping protein localization on polytene chromosomes. The localization of GFP-Tea (**A**) and GFP-Ver (**B**) under different genetic backgrounds was monitored as live GFP fluorescence. At least 50 nuclei were examined for each genotype and all nuclei showed a “present” or “absent” pattern of GFP fluorescence similar to the ones chosen for the Figure. The localization of HOAP and HipHop under similar genetic backgrounds was monitored by antibody staining (**C**). For **A** and **B**, genotypes are indicated at the top left of the images. For **C**, genotypes are indicated to the left of the images. In **A** and **B**, the left panel displays merged images of GFP signal in red and DNA signal in white, and the right panel displays images with GFP signals in white. In **C**, the left panel displays polytene chromosomes in white with telomeres indicated with asterisks. The middle panel displays images with anti-HOAP (**H**) signal in white. The right panel displays anti-HipHop (**HH**) signals. **D**. HOAP (and likely HipHop), Tea, and the Moi-Ver complex are components of a linear pathway for capping protein loading. Binding of capping proteins ultimately controls the accessibility to chromosome ends as suggested by the ExoI protection experiments shown in Fig 4. Genotype of mutants: *ver*^*S147910*^*/Df*; *moi*^*CB02140*^*/Df*; *tea*^*1755*^*/Df*; *cav*^*1*^*/Df* (*Df* is a chromosomal deficiency of the corresponding mutant locus.).

In *tea* mutants, both HOAP and HipHop form foci similarly to wildtype cells (Fig 2C), suggesting HOAP and HipHop localization are minimally affected by the loss of Tea function. Therefore, Tea does not appear to control the localization of the HOAP-HipHop complex. On the contrary, HOAP seems to control Tea’s localization since in *cav* mutants, GFP-Tea fails to form discernable stripes in polytene cells (Fig 2A). We did not investigate Tea’s localization in *hiphop* mutants due to their lethality in the embryonic stage [20]. Interestingly, Ver localization is defective in *tea* mutants as we did not observed GFP-Ver signals in mutant nuclei (Fig 2B), suggesting Ver localization depends on Tea function. However, the converse is not true. In *ver* mutant nuclei, GFP-Tea forms foci that appear normal (Fig 2A). Consistent with previous results, GFP-Ver fails to localize in *cav* mutant cells (Fig 2B, ref [9]). Interestingly, Moi is also dispensable for Tea localization to telomeres (Fig 2A), which is consistent with previous results suggesting that Moi and Ver function in similar fashion [9; 22]. Fig 2D summarizes the functional relationship among known capping proteins established in this and previous studies, which suggest that Tea might functionally regulate Moi and Ver. We then set out to study whether Tea interacts with Moi and Ver as a complex.

### Moi, Ver and Tea physically interact

**Fig 3:**
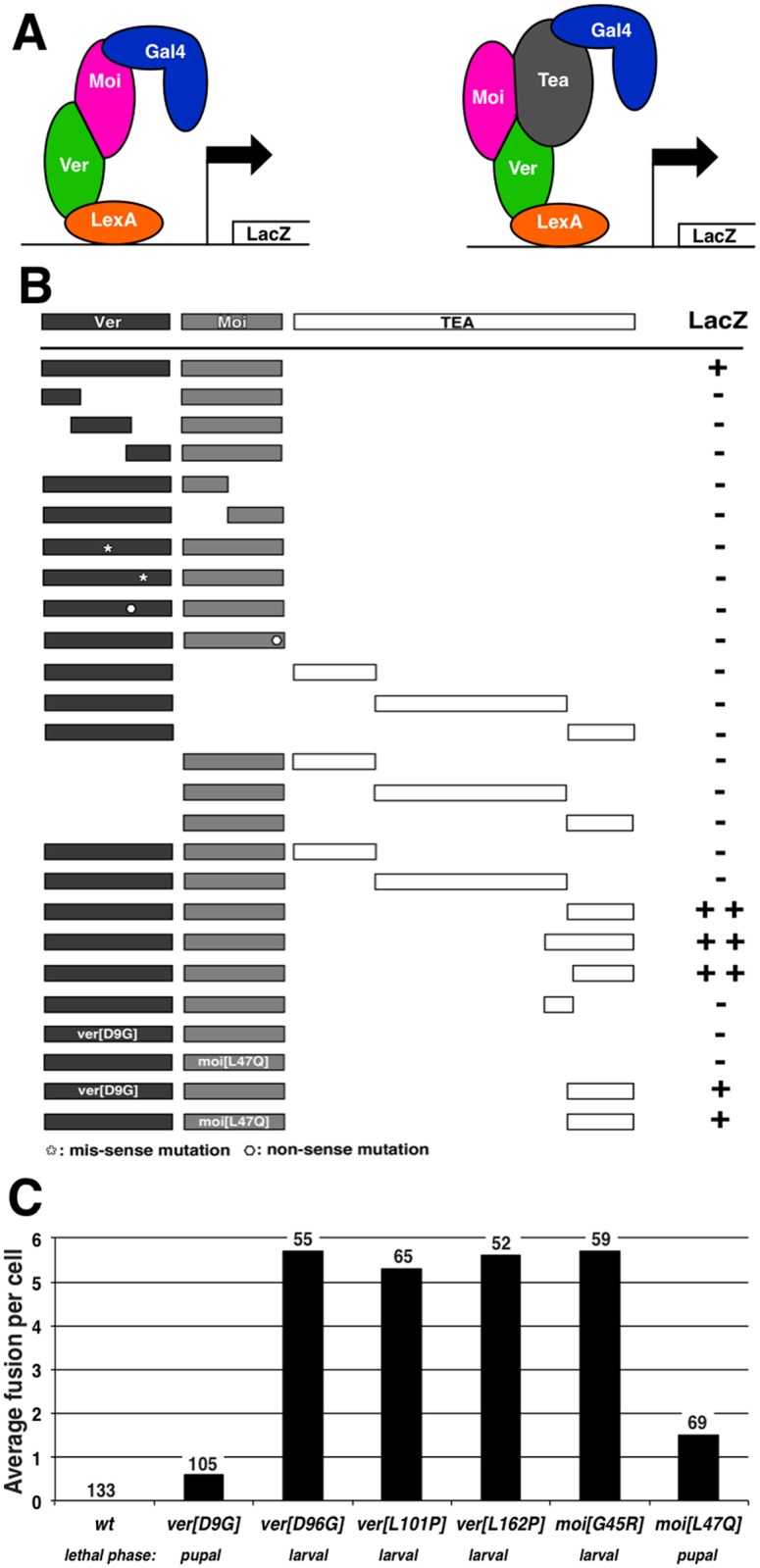
Characterization of MTV interaction with yeast two-hybrid and genetic mutations. **A**: schematic illustrations of yeast two-hybrid (left) and three-hybrid (right) assays. In yeast three-hybrid, either Moi or Ver were expressed as “stand-alone” proteins. **B**: diagrams summarizing the yeast two/three hybrid results. Moi and Ver interact in yeast two-hybrid and Moi, Ver and C-terminal Tea interact in yeast three hybrid. Black and grey rectangular boxes indicate the extent of Ver and Moi proteins expressed respectively. We generated point mutations in *ver* and *moi* to disrupt MV interaction (see main text), which are indicated as white dots and asterisks. The white rectangular boxes represent the fragments of Tea expressed in yeast. **C**: a chart showing the extent of telomere uncapping in *ver* and *moi* point mutants identified in the yeast two-hybrid screen. The frequencies of telomere fusion are shown for each mutant with the number of nuclei scored (n) listed on top of the bars. The amino acid changes in each mutant are indicated in brackets. The lethal phase of these mutants are indicated at the bottom.

Ver has been shown to share sequence similarity with Stn1 from organisms with telomerase-maintained telomeres [9; 11]. In these organisms, Stn1 forms a complex with the Ten1 protein. We reasoned that if a similar complex exists in Drosophila, Moi is a likely candidate for forming a complex with Ver. Indeed, Raffa et al. [9] obtained evidence suggesting that Moi and Ver interact *in vitro*. To further establish physical interaction between Ver and Moi, we employed the yeast two hybrid (Y2H) assay, the most commonly used assay in the study of Stn1-Ten1 interaction. As shown in Fig 3B, Ver and Moi interact in Y2H. Interestingly, even small truncations of either Ver or Moi disrupted interaction. Although this suggests that both Ver and Moi function as a single domain protein, we cannot rule out that the negative Y2H results were due to the truncated proteins being unstable when expressed in yeast. Ver and Moi also interact when co-expressed in insect cells (see later). But to further strengthen the functional significance of a Moi-Ver complex, we sought to test *in vivo* the consequence of disrupting Ver-Moi interaction with single residue missense mutations. We suspected that such point mutations should have severe functional consequences possibly leading to the complete loss of function of both proteins.

We conducted a small-scale random mutagenesis screen to find amino acid residues in both Ver and Moi that are essential for their interaction based on the Y2H assay (Supplemental Materials) and were able to recover missense mutations that disable interaction. We chose four *ver* (D9G, D96G, L101P and L162P) and two *moi* mutations (G45R and L47Q), and introduced them into the endogenous loci using SIRT. All except one *ver* and one *moi* mutations behaved as null mutations judged by comparing the lethal phases and telomere fusion frequencies in these mutants with known null mutations (Fig 3C). The two exceptions will become interesting once we discuss our results from studying the interaction between Tea and Moi or Ver. Therefore, our Y2H and genetic results strongly suggest that Moi and Ver form and function as an MV complex *in vivo*.

Since Tea is a rather large protein, predicted to be close to 1900 residues (flybase.net), we arbitrarily divided it into three fragments in Y2H studies. To our surprise, none of the Tea fragments interacted with Moi or Ver alone (Fig 3B). We reasoned that if Moi, Tea and Ver were to form a complex, all three members might need to be present to establish stable interaction. To detect such interactions with Y2H, we modified the standard set up into a “yeast 3-hybrid” (Y3H) assay in which (1) Tea fragments were expressed as fusions to the activation domain of Gal4; (2) either Ver or Moi was expressed as fusions with the DNA binding domain of LexA; and (3) either Moi or Ver was expressed as a third but stand-alone protein (Fig 3A). With this Y3H set up, we were able to detect strong interactions of Moi, Ver and the C-terminus of Tea (Fig 3B). In addition, this tripartite interaction is disrupted by either Moi or Ver truncations. Interestingly, four of the six missense *ver* or *moi* mutations that we recovered from the Y2H-based mutant screen also disrupt Moi-Tea-Ver interaction. For the two exceptions (*ver*^*D9G*^ and *moi*^*L47Q*^), Moi-Ver interaction in Y2H was disrupted, yet restored significantly by the presence of C-terminus of Tea in Y3H. Remarkably, these two mutations behave as partial loss of function mutations when introduced into flies (Fig 3C). We suggest that the loss of Moi-Ver interaction caused by *ver*^*D9G*^ and *moi*^*L47Q*^ mutations was also partially restored *in vivo*, which lends strong support to the hypothesis that Moi, Tea and Ver form and function as a complex in Drosophila, which we name the MTV complex.

### A recombinant MTV complex binds and protects ssDNA

The CST or TPP1-POT1 complexes bind single stranded DNA (ssDNA) *in vitro*. We speculated that MTV possesses similar ssDNA binding activity and set out to investigate this possibility with recombinant MTV subunits.

We were able to recapitulate the MTV interaction observed in Y3H using MTV proteins individually expressed and purified in bacteria (S2A Fig in Supplemental Materials). However, it has been technically difficult to express and purify sufficient amount of full length Tea protein in bacteria for testing ssDNA binding. We turned to the baculoviral system using insect cells and expressed all three subunits simultaneously but as differently tagged proteins: V5-Moi; 3xFLAG-Ver and 3xHA-Tea. We also expressed just the V5-Moi and 3xFLAG-Ver proteins in insect cells to study the property of the MV sub-complex. Through a single anti-FLAG purification, we were able to significantly purify the MTV as well as the MV complexes from insect cells (S2B Fig). Further precipitation with either anti-HA (for Tea) or anti-V5 (for Moi) did not result in improved purification of MTV. Therefore, we conducted subsequent DNA binding assays with this partially purified MTV complex.

**Fig 4:**
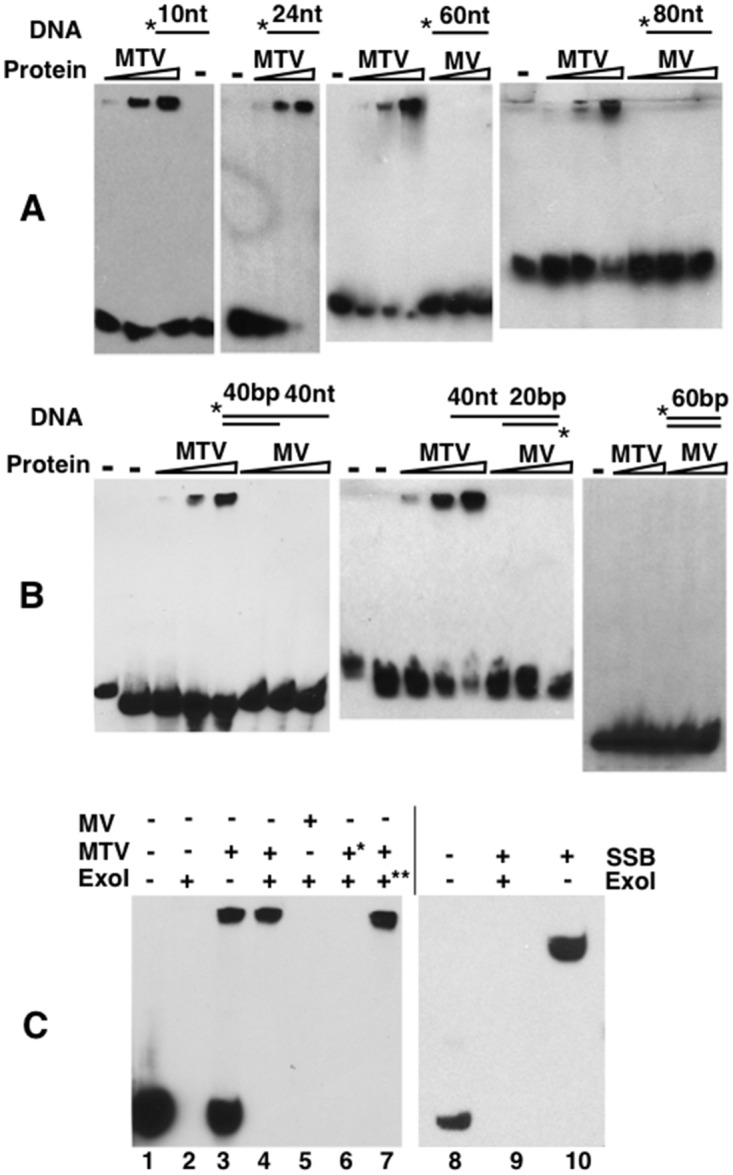
MTV binds and protects ssDNA. **A**: MTV but not MV binds ssDNA. Increasing amount of MTV or MV were mixed with 5’-labelled oligos (*). The oligo is shown as a line with size indicated on top. **B**: MTV binds single stranded overhangs of dsDNA. The overhangs are indicated on top of each image with the size of the double stranded and single stranded regions indicated on top of the double line. Free single stranded oligos with the 5’-end label were loaded in the first lanes of the left and middle images. In the second lane of these images, the annealed products of the two oligos were loaded and ran. **C**: MTV, not MV or bacterial SSB, protects ssDNA from bacterial ExoI. For lanes 4 and 6, MTV and ExoI were mixed sequentially with the oligos. But for lane 6 (*), MTV was first boiled for 5 minutes before mixing. For lane 7 (**), MTV and ExoI were first mixed then added to the binding reaction with the oligos.

As shown in Fig 4A, recombinant MTV binds ssDNA as short as 10 bases. In contrast, dsDNA as long as 60bp could not elicit MTV binding, which suggests that MTV specifically binds ssDNA. Binding of MTV was also observed with longer oligos (up to 84 bases that we tested). Since all the oligos we used in testing MTV binding are unrelated in sequence, we conclude that MTV binding to ssDNA is sequence independent. Interestingly, an MV complex purified in parallel did not bind either ssDNA or dsDNA under our experimental conditions (Fig 4A). As telomeres in many organisms end in a 3’ overhang, we investigated whether a 3’ single stranded end is needed for MTV binding. We provided the binding substrates as two annealed oligos with different lengths so that either a 5’ or a 3’ overhang remained. As shown in Fig 4B, MTV binds both substrates suggesting that a 3’ end is not absolutely required for MTV binding *in vitro*.

We were interested in whether MTV binding to ssDNA confers any protection to the ends and developed an assay based on the exo-nuclease activity of the bacterial ExoI enzyme. Under our experimental conditions, ExoI efficiently degrades the labeled oligos (Fig 4C). However, the oligos were effectively protected in the presence of MTV. MTV did not confer protection by poisoning ExoI since only the MTV-bound oligos were protected (Fig 4C lane 4). In addition, pre-incubation of MTV with ExoI did not qualitatively alter the ability of MTV to bind and to protect oligos nor ExoI’s ability to degrade un-bound oligos (Fig 4C lane 7). This protection appears specific to MTV since the protection cannot be rendered by either the MV complex or the bacterial ssDNA-binding protein SSB, which nevertheless binds ssDNA efficiently in the absence of ExoI (Fig 4C lane 10). Therefore, MTV specifically binds ssDNA and protects ssDNA against exo-nucleolytic degradation.

## Discussion

We have been characterizing proteins essential for the protection of telomeres in Drosophila, an organism that fascinates the field as one that lacks the telomerase enzyme. Here by characterizing the newly identified Tea protein and its associated factors, we showed that Tea physically and functionally interacts with two other capping proteins, Moi and Ver, forming the MTV complex. A purified MTV binds single stranded oligos *in vitro*, and remarkably, binding of MTV confers resistance to nucleolytic degradation to ssDNA. This mode of protection is reminiscent of the *in vivo* situation in which the binding of capping proteins to chromosome ends shields them from degradation and DNA repair activities. In previous reports [23; 24], purified yeast Cdc13 and mammalian POT1 confer similar ssDNA protection suggesting that telomeric ssDNA-binding proteins from diverse systems have fundamentally conserved molecular and functional characteristics.

It would be interesting to determine the division of labor among the three subunits of MTV with regards to ssDNA binding and/or protection. In particular, the larger Tea warrants a finer structural dissection in which functional domains of the protein might be identified, similarly to what has been done for the yeast Cdc13 protein [reviewed in 25]. The fact that Tea is able to localize to telomeres even in the absence of Moi or Ver suggests that Tea alone has the ability to bind ssDNA, a hypothesis that we are actively testing. Nevertheless, Tea’s localization to telomeres in *moi* or *ver* mutants does not have to be via direct binding to ssDNA, but rather via interaction with other components of the telomeric chromatin.

Our genetic analyses on the inter-dependence of capping protein localization suggest a linear pathway (Fig 2D). Based on this model, we envision the sequence of possible molecular events that have to occur at Drosophila telomeres for their proper protection. The HOAP-HipHop complex occupies a large genomic region at chromosome ends, as we have previously shown [6]. This might help establish a favorable chromatin configuration for the recruitment of Tea to the ssDNA overhang. Binding of Tea in turn enables the recruitment of the Moi-Ver sub-complex.

The binding of MTV prevents the degradation of ssDNA by bacterial ExoI enzyme *in vitro* (Fig 4C). Since that ExoI removes ssDNA in a 3’ to 5’ direction and that telomeric overhangs are mostly 3’ in nature, telomeric binding of MTV might function to prevent the loss of telomeric overhangs. However, the nuclease activities *in vivo* are highly complex, it would be premature to suggest that loss of MTV would lead to overhang shortening based on our *in vitro* data. Instead, we suggest that our *in vitro* results shown in Fig 4 are consistent with the model in which the function of MTV binding at telomeres is to regulate accessibility to chromosome extremities.

Although our *in vitro* results suggest that MTV binds ssDNA, we have yet to provide direct evidence confirming that Drosophila telomere ends in an overhang as our attempts have not generated convincing results so far. We believe that several intrinsic properties of telomeric DNA in Drosophila have made it technically challenging to detect overhangs. Current overhang detection methods take advantage of the highly repetitive nature of the telomeric sequences in telomerase-based organisms [e.g. 26–28]. Because of this feature of telomerase-added telomeres, sequences at the very terminus are known and all telomeres in a cell end in essentially identical sequences. This aids the design of probes or PCR primers for overhang detection. Drosophila telomeres, on the other hand, consist of intermediately repetitive sequences from three different transposons, with each repeat unit being several kilobases in length. Therefore, the terminal sequences in Drosophila vary greatly and quite possibly are never the same on more than one telomeres. Therefore, using natural transposon sequences as starting point for overhang detection is unproductive. Nevertheless, Drosophila telomeres are likely to end in an overhang considering that: (1) after a round of replication the lagging strand telomere ends in an overhang after removal of the RNA primer; and (2) the enzymes so far identified as important for overhang processing have homologous proteins in Drosophila. Therefore, our identification of MTV as an ssDNA-protecting complex should have biological relevance for Drosophila.

Here MTV and CST complexes were frequently discussed in parallel, however, we do not intend to suggest that MTV is structurally analogous to CST. Although CSTs from different organisms share a common name, its subunits are known to be remarkably diverse. For examples, fungal Cdc13 proteins can vary greatly in size [e.g. 29]; Stn1 from Arabidopsis might be too small to contain a WH domain present in fungal Stn1 proteins, which is important for interacting with Cdc13 [reviewed in 10]. Therefore, even though CST complexes share similar biological functions in different organisms, their modes of interaction and even complex composition can be remarkably diverse. For this latter point, the stoichiometry of CST in Candida was recently determined to be 2:4:2 or 2:6:2 rather than 1:1:1 [30]. Therefore, proving a complex is structurally analogous to CST is not trivial in any system, let alone in Drosophila where the protein divergence seems to be at the extreme. It is also worth noting that the ability of MTV to bind ssDNA in a sequence non-specific fashion resembles that of the RPA complex essential for genome replication. In fact the CST complex has been described as a telomeric RPA-like complex based on structural similarities of the two [31; 32]. In addition, CST in mammals is important not only for telomere maintenance but also for genomic replication [33]. Whether the Drosophila MTV complex has a similar role in regulating genome-wide replication remains to be studied.

Our work and that of others continue to reveal remarkable conservations in both the biological features of chromosome ends and the protein factors that regulate their function between the Drosophila system and those involving telomerase. In addition, Drosophila studies have yielded insights into telomere functions that are novel even for telomerase-maintained systems. In one example, we and others showed that a paternally installed protein imprint on sperm telomeres protects them from DNA repair activities in early embryos [20; 34]. It is difficult to envision that paternal telomeres enter the egg as “naked” DNA in any animal species. Therefore a similar paternal imprint likely exists universally. The fact that the Drosophila imprint consists of the K81 protein, a sperm specific variant of HipHop, suggests that the mammalian imprint could also be a component of the duplex binding complex. It would be interesting to investigate whether the newly identified ssDNA-binding MTV complex participates in the maintenance of this paternal imprint.

## Materials and Methods

### Drosophila genetics

All stocks were obtained from the Bloomington Drosophila stock center unless noted otherwise. All stocks are described in flybase (flybase.net) unless noted otherwise.

### Gene targeting in Drosophila

Epitope tags and point mutations were introduced into the endogenous loci of *moi*, *tea* and *ver* using the SIRT gene targeting method that we developed for Drosophila [19]. Detailed experimental designs are presented in Supplemental Materials. Briefly, an *attP* landing site for the phiC31 integrase was first introduced to the vicinity of the locus of interest by traditional gene targeting by homologous recombination [35; 36]. A plasmid containing the desired modification, a tag or a mutation, was integrated into the locus of interest by phiC31-mediated site specific integration followed by I-CreI endonuclease-induced reduction of the target locus duplication to generate a final locus containing the desired modification.

### Yeast two-hybrid assay

LacZ-based yeast two hybrid assays were performed as described using an identical set of vectors and yeast strains [32]. We modified the traditional yeast two-hybrid assay into a three-hybrid assay in order to detect tripartite interaction involving all three subunits of MTV. Detailed protocols are given in Supplemental Materials.

### Recombinant protein purification from insect cells

MTV expression in insect cells was based on the Bac-to-Bac Baculovirus Expression system from Invitrogen. The correct DNA clones containing 3xFLAG-Ver, 3xHA-Tea and V5-Moi were confirmed by sequencing. Baculovirus were generated by the service of the Protein Expression Laboratory (PEL) of NCI, Frederick with the titer above 1x10^8^ pFU/ml. Detailed protocols for protein expression and purification are provided in Supplemental Materials. Between 50ng to 200ng of purified MTV and 100ng to 400ng of purified MV were used for EMSA.

### EMSA assay

Protein-oligo interaction was analyzed using LightShift Chemiluminescent EMSA Kit (Pierce) according to the manufacturer protocols using 5’ Biotin-labeled oligos synthesized by Integrated DNA Technologies. The ExoI protection assay was performed in the same binding buffer with 2U of the enzyme from NEB and incubated for 30 minutes at RT.

### Cytology

Telomeric localization of Tea, HipHop and HOAP by antibody staining was performed as described in ref [6]. A rabbit anti-GFP antibody from Torrey Pine Biolabs was used to localize Tea at polytene telomeres using published protocols. For live GFP observation, tissues were dissected in PBS with 0.1% Triton X-100 (PBST), transferred to a DAPI solution in PBST. The tissues were stained for five minutes followed by a wash in PBST, and mounted in PBS.

## Supporting Information

S1 Text Detailed descriptions of Materials and Methods and legends for supplemental figures. (DOCX)

S1 Fig Genomic structure of the mtv loci. (TIFF)

S2 Fig A partial purification of the MTV complex. (TIF)

S1 Table List of primers used and their sequences. (PDF)
