# Supplementary material for: MTV, an ssDNA Protecting Complex Essential for Transposon-Based Telomere Maintenance in Drosophila
Source: PLoS Genet. 2016 Nov 11;12(11):e1006435. doi: 10.1371/journal.pgen.1006435 (PMC5105952; doi:10.1371/journal.pgen.1006435)
Supplement: S1 Text — (DOCX) [file pgen.1006435.s001.docx]

**Supplemental Text**

**MTV alleles**

*Construction of a tea rescue plasmid and its derivatives.* The genomic region of the *tea* locus is depicted in S1 Figure. A 10kb genomic fragment of *tea* from nucleotide nt9890613 to nt9900409 on chromosome 2R (flybase), which includes about 1.5kb of upstream and 2kb downstream elements of *tea*, was subcloned from a genomic BAC clone (BACR30G11) into the gene targeting vector pTV2 [1] using gap-repair by recombineering method [2; similar methods have been described in details in 3]. This gave rise to pTV2[tea], which was subsequently introduced into the fly genome by P element mediated transformation. This construct was able to rescue viability to all *tea* mutant allelic combinations. Using recombineering designs described in ref 4, we placed an *egfp* gene just downstream of the start codon of *tea* (S1 Figure) and generated pTV2[GFP-tea]. This construct was also able to rescue all *tea* mutant combinations confirming the functionality of the *gfp*-tagged *tea* locus. For conducting SIRT gene targeting [5; 6], we placed an *attP* landing site for the phiC31 integrase between nt9898741-2, 3’ of the coding region, and subsequently introduced the N-terminal *egfp* tag to the endogenous *tea* locus. Flies with this *egfp-tea* as the only *tea* locus are viable and fertile, and are used in *tea* localization studies.

*Tea mutant alleles*. We sequenced the coding region in the previously isolated *tea^1755^* mutant and identified a mutation changing CAG to a premature stop codon TAG (Q^1638^>stop, S1 Figure) as well as several amino acid residue differences between *tea^1755^* and the annotated *tea* sequence (flybase.net). We also obtained the P element insertion line *l(3)SH0071* [7]. Trans-heterozygotes of *l(3)SH0071* with either a *tea* deficiency (*df-tea*) or *tea^1755^* are semi-lethal with few adult survivors. This partial lethality is rescued by the pTV2[tea] construct, suggesting that *l(3)SH0071* is a hypomorphic mutation of *tea* consistent with it having a P element inserted 5’ and outside of the *tea* coding region. We rename *l(3)SH0071* as *tea^SH0071^*. During the placement of *attP* at *tea* during SIRT gene targeting, we recovered another *tea* allele that we name *tea^2-1^*. Homozygotes of *tea^2-1^* and trans-heteroygotes of *tea^2-1^* with *df-tea* or *tea^1755^* are strictly larval lethal with telomere fusions in larval neuroblasts. These defects are fully rescued by pTV2[tea]. *tea^2-1^* might belong to a class III gene targeting event [1; 8] in which both copies of the *tea* gene are disrupted by small rearrangements during the homologous recombination process.

*ver and moi alleles*. Using a combination of bacterial recombineering and SIRT gene targeting in Drosophila [4; 6], we placed an *attP* landing site 5’ to *ver* or 3’ to *moi* coding regions. The *attP* at *ver* is between nt12524212-3 of chromosome 3L and *attP* at *moi* is between nt18257779-80 on chromosome 3R (flybase.net). Using these landing sites, we placed a *gfp* tag N-terminal to the endogenous *ver* locus for monitoring Ver localization. The *ver* and *moi* point mutations recovered from the yeast 2 hybrid based screen were introduced similarly using *attP* landing sites as described previously [4].

**Yeast genetics and molecular biology**

*Saccharomyces cerevisiae* strain CTY10-5d (MATa ade2 trp1-901 leu2-3,112 his3-200 gal4-gal80-URA3∷lexA-lacZ) contains an integrated GAL1-lacZ gene with the lexA operator (a gift from Dr. Ming Lei at University of Michigan) was used for all yeast related experiments.

*Yeast Transformation.* CTY10-5d was incubated at 30°C overnight (O/N) in YPD medium. The next day, 2 ml of culture was transferred to 20 ml of fresh YPD and incubated until OD600=1 (~ 4 h). The cells were pelleted at 800g for 5 min, re-suspended in 1 ml autoclaved water, and pelleted again. The cell pellet was re-suspended in 1 ml TE-Li buffer (2.7 mL 1XTE buffer and 0.3 mL 1M Li-Acetate), and pelleted. The cell pellet was re-suspended into 0.3 mL TE-Li buffer and used for transformation. For transformation, 1 µl of LexA containing and GAL4 containing plasmids (~100ng each) were mixed with 25 µl competent yeast cells, 25 µl sperm DNA (2mg/ml) then 5x volume of filtered 40% PEG (Sigma, Cat#: p4338) in Li-TE buffer were added to the mixture. The mixture was incubated in 30°C for 30 min, 2.5 µl of DMSO was added, and then a 10 min heat shock was given at 42°C. Mixture was put on ice, and 0.6 ml of sterile H2O was added. Cells were spun down at 1000g for 1 min, re-suspended in 100 µl of H2O and plated on CSM-Leu-Trp plates.

*DNA extraction from yeast.* Single yeast colony was suspended in 150 µl of TE buffer, then 150 µl of PCI (Phenol:Chloroform:IAA) was added, and ~300 µl of glass bead (Sigma) was added . The mixture was shaken vigorously for 20-30 min, and spun down at 14000 rpm for 15 min. The supernant (~50 µl) was collected and 0.5-2 µL was used for PCR.

*Protein extraction from yeast.* 5 ml of yeast culture was grown to OD600=~2.5 and cells were spun down at 5000g for 3 min, then re-suspended in 100 µl of distilled water. 100 µL of 0.2M NaOH was added, and the sample was incubated at room temperature for 5 min. The sample was pelleted, re-suspended in 50 µl of PAGE sample buffer, boiled for 3 min, and pelleted again. The supernatant was collected for gel electrophoresis and Western blot analyses.

**Yeast two-hybrid and three-hybrid assays for MTV interaction**

*Yeast-2-Hybrid*. The plasmid pBTM116 was used to construct fusion proteins of LexA-proteins of interest. The plasmid pACT2 was used to generate fusion proteins of GAL4AD-proteins of interest. The protein of interest was cloned into the MCS to make sets of vector, pBTM-Ver, pBTM-Moi, pACT-Ver, pACT-Moi, pACT-TEA-N, pACT-TEA-M, pACT-TEA-C, pACT-Ver-N, pACT-Ver-C, pACT-Moi-N and pACT-Moi-C. Primers used to PCR the inserted genes are showed in Table S1. Each pair of plasmids encoding a LexA-fusion of protein of interest and a GAL4AD-fusion of protein of interest was co-transformed into yeast strain CTY10-5d. Three pairs of plasmids were used as negative controls: empty vectors of pBTM116 and pACT2, or empty pBTM116 with pACT2 containing GAL4AD-fusion of protein of interest, or empty pACT2 with pBMT116 containing LexA-fusion of protein of interest. Transformants were selected on CSM-Leu-Trp plates. Every single colony was re-plated on CSM media containing X-gal (0.1mg/ml X-Gal in Dimethylformamide). The blue (lacZ expressing) or white color of colonies was determined one week later.

*Yeast two-hybrid based screen for mutations disrupting Moi-Ver interaction.* To generate a library of *moi* or *ver* genes carrying random mutations, we first convert the pBTM116 vector into one compatible with the Gateway cloning technology. This was accomplished by ligating the Frame B cassette from the Gateway Vector Conversion system from Invitrogen into a SmaI digested pBMT116. The cassette carries the *cmR* and *ccdB* genes permitting both positive and negative selections with *cmR* and *ccdB* respectively. This generates pBMT-cmR-ccdB. The cmR-ccdB cassette is flanked by attR1 and attR2 sites for gateway manipulation. Frame B was chosen to ensure LexA and the following Moi or Ver proteins are in frame.

The *moi* and *ver* mutant libraries were generated by error-prone PCR using the Diversify PCR Random Mutagenesis kit from Clontech using a predefined condition that would generate an average of two nucleotide changes per one kb of amplification (both *moi* and *ver* coding regions are less than one kb). The DNA products in this PCR amplified library carry flanking attL1 and attL2 sites, which were then used to replace the cmR-ccdB cassette in pBMT-cmR-ccdB. This generated a pBMT-based library of potential *moi* and *ver* mutations. Individual pBMT plasmids were co-transformed with a pACT-based plasmid carrying the wildtype coding region for the interacting protein. Yeast transformants were tested for lacZ expression normally. Plasmid DNA was recovered from colonies that remain white after one week and the pBMT-based plasmid was retested with a new pACT-based plasmid in yeast 2 hybrid. For clone that passed the second test, sequencing was perform to identify the causative mutation(s).

To exclude that a point mutation destabilizes the protein to produce the negative yeast 2 hybrid results, we recovered total extracts from white colonies of the yeast 2 hybrid assay, and performed Western blot analyses using an anti-LexA antibody from Abcam since the mutant Moi or Ver proteins have an N-terminal LexA fusion. The control was extracts from blue colonies with plasmids containing withtype genes. In all cases tested, the LexA fusion proteins, either wildtype or mutant, were expressed as similar levels.

*A modified yeast two-hybrid assay (yeast 3-hybrid).* To study interaction among Moi, Ver and Tea proteins, we modified the traditional yeast two-hybrid assay. The new assay was based on three plasmids: pBMT-Moi, pBMT-Ver and pACT-Tea. The pBMT plasmids provide expression of Moi and Ver fused with the DNA binding domain of the LexA protein. These plasmids confer Trp+ to trp-mutant yeast cells. The pACT-Tea plasmid allows expression of Tea fragments fused to the activation domain of Gal4, and it confers Leu+ to leu-mutant yeast cells. We first replaced the LexA coding region in the two pBMT plasmids with the cmR gene using recombineering with the following primers: pBTM-CmnoLex-F and pBTM-Cm-Moi-R for Moi; pBTM-CmnoLex-F and pBTM-Cm-Ver-R for Ver as listed in Table S1. This cmR gene was subsequently removed by restriction digest followed by intra-molecular ligation. This generated pFree-Moi and pFree-Ver plasmids, which allow expression of LexA-free Moi and Ver proteins respectively. The two new plasmids were further modified by replacing the Trp+ marker with KanMax by recombineering, which confers G418 resistance to yeast cells, using the following primers: pBTM-CmKm6-F and pBTM-CmKm6-R. This generates pFree-Moi-Km and pFree-Ver-Km. Three plasmids, one pBMT based, one pFree-Km based, and pACT-Tea were transformed into yeast cells by selection for Trp+ Leu+ and G418R (250mg/ml final concentration). Triple positive cells were assayed for LacZ expression as in the normal yeast two-hybrid assay.

**Recombinant protein expression from *E.coli***

Moi was cloned into plasmid pQE30 (Qiagen) with primers QE-SphI-Moi-up and QE-Moi-HindIII-down. Protein was expressed in E.coli M15 cells. The Flag tagged Ver was cloned in plasmid pET28 with primers pET-NdeI-Verflag and pET-EcoR-Ver-R, and protein was expressed in E.coli BL21 cells. TEA-N or TEA-C was cloned in plasmid pET28 with primers pET-NdeI-1f and pET-XhoI-348r or pET-NdeI-1584f and pET-XhoI-1876r, respectively. The V5 tag was added in the N-terminus with primers V5-AscI+Cm and Ascl+Cm by using recombineering technology. Proteins were expressed in E.coli BL21 cells. Cell pellets were sonicated in RIPA buffer (50 mM Tris, pH7.6, 150 mM NaCl, 30 mM KCl, 1% NP-40) with proteinase inhibitor cocktails (Roche) and Bezonase (2U/ml of the final concentration, Sigma). The lysates were centrifuged for 30min at 20000g. The supernatants were collected and equal volumes of three extracts were mixed. Co-immunoprecipitation was performed using 0.5 ml of mixed extracts and anti-V5 antibody (ab9116, Abcam) to pull-down, then detected by anti-His (Qiagen), anti-Flag (Sigma) and anti-V5 (Abcam) individually.

**Recombinant protein expression and purification from insect cells**

*Expression vectors construction.* MTV expression is insect cells are based on the Bac-to-Bac Baculovirus Expression system from Invitrogen. pFASTBacHTa was used to express V5-tagged Moi and 3XFLAG-tagged Ver. pFASTBacHTb was used to express 3XHA-tagged Tea. To construct a full-length cDNA clone of Tea, two partial cDNA clones, AY058576 and AY058602, were combined by molecular cloning.

To construct Bacmids for generating baculovirus, plasmids pFastBacHTb-3xHA-TEA, or pFastBacHTb-3xFlag-Ver, or pFastBacHTb-V5-Moi, were transformed separately into MAX Efficiency DH10Bac Chemically Competent E. coli cells, and selected on LB plates containing 50 µg/ml of km, 7 µg/ml of G418, 10 µg/ml of Tet, 100 µg/ml of Bluo-gal (Invitrogen, dissolved in DMSO for 20 mg/ml stock) and 40 µg/ml of IPTG. White colonies (10 of each) were selected and grown in liquid LB medium containing antibiotics at 30°C for O/N. Bacmids were purified using the PureLink HiPure Plasmid DNA Miniprep Kit (Invitrogen) according to the manufacturer protocols. The correct clone was confirmed by sequencing. Baculovirus were generated by the service of the Protein Expression Laboratory (PEL) of NCI, Frederick with the titer above 1x10^8^ pFU/ml.

*Recombinant protein expression.* Sf9 insect cells (Invitrogen) were grown in serum-free medium Sf-900™ II SFM (Invitrogen) at 27°C in a flask shaking at 145 rpm. The day before the infection, cells were seeded in fresh Sf-900™ II SFM at a density of 1x10^6^ cell/ml. Baculovirus expressing 3xHA-TEA, 3xFlag-Ver and V5-Moi were co-infected into Sf9 cells 24 h later. The MOI is 2:2.5:20 for Ver:Moi:TEA. FBS (0.4%) was added into media 5h later, and cells were collected after 3 days of culturing, and stored at -80°C until use.

*Recombinant protein purification*. Cell pellet from a 200ml of culture co-expressing either Moi and Ver proteins or Moi, Ver and TEA proteins was thawed on ice. RIPA (30 ml) buffer containing proteinase inhibitor cocktails (Roche) and Bezonase (Sigma, 2U/ml final concentration) was added to each pellet. Cells were lysed using a Sonicator (Misonix, Inc) for 3 min. at 15 sec ON and 30 sec OFF cycles. The lysates were centrifuged for 1h at 20000g. Supernatants were collected and incubated with 0.25 ml of anti-Flag beads (Sigma) for 3 -4 hours at 4°C. The beads were washed with 20 ml of RIPA buffer, and then eluted with RIPA buffer containing 100 µg/ml of 3xFlag peptide (Sigma) using gravity flow. Each eluted fraction was checked on SDS-PAGE gel, and the fraction containing the target proteins was pooled and concentrated to 200µl. This 200µl was loaded on the top of a 5cm x 1 cm glycerol gradient (5 ml) of 10-40% glycerol in 1x TBS buffer plus 0.02% NP-40, and further separated by centrifuge at 35000g for 16 hours at 4°C. The glycerol gradient was divided into 25 fractions and each fraction was checked on SDS-PAGE gel. The purified protein was pooled and passed through the PD10 column (GE) to de-salt with 1XPBS buffer, and then stored at -80C until use.

**EMSA Assay**

Protein-oligo interaction was analyzed using LightShift Chemiluminescent EMSA Kit (Pierce) according to the manufacturer protocols with slight modifications. The binding reaction includes 2 µl of 10x binding buffer, 1 µl of each of polyI/C, glycerol, KCl, MgCl2 and NP-40 from the kit, 2 µl of 2 nM biotin-labeled oligos (synthesized by IDT) and the tested protein in a total volume of 20 µl. Between 50ng to 200ng of purified MTV and 100ng to 400ng of purified MV were used for EMSA. The reactions were analyzed by running 5.5% native polyacrylamide gel, and signals were detected by following the protocol for the LightShift Chemiluminescent EMSA Kit (Pierce). Native polyacrylamide gels were prepared with: 5.5 ml of 30%:0.8% Acrylamide/Bis 37.5:1 (Bio-Rad), 24.5 ml of 0.2x TBE buffer (KD Medical), 150 µl of APS (Bio-Rad) and 30 µl of TEMED (Bio-Rad). Gel was pre-run to clean the wells for 0.5 hours before sample loading. The ExoI protection assay was performed in the same binding buffer with 2U of the enzyme and incubated for 30 minutes at RT.

**References**

5. Gao G, McMahon C, Chen J, Rong YS. A powerful method combining homologous recombination and site-specific recombination for targeted mutagenesis in Drosophila. Proc Natl Acad Sci U S A. 2008;105:13999-14004.

6. Gao G, Wesolowska N, Rong YS. SIRT combines homologous recombination, site-specific integration, and bacterial recombineering for targeted mutagenesis in Drosophila. Cold Spring Harb Protoc. 2009;2009(6):pdb.prot5236.

7. Oh SW, Kingsley T, Shin HH, Zheng Z, Chen HW, Chen X, Wang H, Ruan P, Moody M, Hou SX. A P-element insertion screen identified mutations in 455 novel essential genes in Drosophila. Genetics. 2003;163: 195-201.

8. Rong YS, Golic KG. Gene targeting by homologous recombination in Drosophila. Science. 2000;288: 2013-2018.

1. Rong YS, Titen SW, Xie HB, Golic MM, Bastiani M, Bandyopadhyay P, et al. Targeted mutagenesis by homologous recombination in D. melanogaster. Genes Dev. 2002;16: 1568-1581.

2. Sharan SK, Thomason LC, Kuznetsov SG, Court DL. Recombineering: a homologous recombination-based method of genetic engineering. Nat Protoc 2009;4: 206–223.

3. Wesolowska N, Rong YS. Long-range targeted manipulation of the Drosophila genome by site-specific integration and recombinational resolution. Genetics. 2013;193: 411-419.

Zhang Y, Schreiner W. Rong YS.. Genome manipulations with bacterial recombineering and site-specific integration in Drosophila. Methods in Molecular Biology 2014;1114: 11-24.

**Figure Legends**

**S1 Figure. Genomic structure of the *mtv* loci.**

Black boxes represent coding regions with the arrow head indicating the direction of transcription. The approximate locations of the N-terminal *gfp* tag and the *attP/attB* landing sites are shown. The approximate position of the Q^1638^ to STOP mutation in the *tea^1755^* allele is shown. The 10kb *tea* genomic fragment was introduced by *P* element-mediated transformation. We recovered three independent transformant lines for *pTV[tea]* and four for *pTV[egfp-tea]*. Lines with the constructs inserted onto chromosome III (one for *pTV[tea]* and two for *pTV[egfp-tea]*) were used to introduced into various *tea* mutant combinations by crossing. All three were able to rescue viability and fertility to *tea* mutant flies. Flies with the only wildtype *tea* locus located on the *P* element were kept as stocks.

**S2 Figure. A partial purification of the MTV complex.**

**A**: interaction among bacterially expressed MTV proteins. Epitope-tagged proteins purified from bacteria are indicated on top to the right. The top images show anti-His Western blot analyses with lanes 5 and 6 had samples from an anti-V5 IP to recover Tea-interacting proteins. Both Ver and Moi were pulled down with Tea-C but not with Tea-N. In lanes 7 and 8, the same samples of lanes 5 and 6 were probed with anti-V5 (top) and anti-FLAG (bottom) antibodies to identify Tea fragments (V5-tagged) and Ver (FLAG-tagged). **B**: the MTV complex purified from insect cells. The image on the left is of coomassie staining of a protein gel with samples from anti-FLAG IP of baculoviral extracts expressing Moi and Ver (left lane) or Moi, Tea and Ver (right lane). The sizes of the molecular markers are indicated to the right. The right three images are Western blots of the same samples as the left gel and probed with the antibodies indicated on the top.
