# Supplementary figures and images for: MTV, an ssDNA Protecting Complex Essential for Transposon-Based Telomere Maintenance in Drosophila
Source: PLoS Genet. 2016 Nov 11;12(11):e1006435. doi: 10.1371/journal.pgen.1006435 (PMC5105952; doi:10.1371/journal.pgen.1006435)

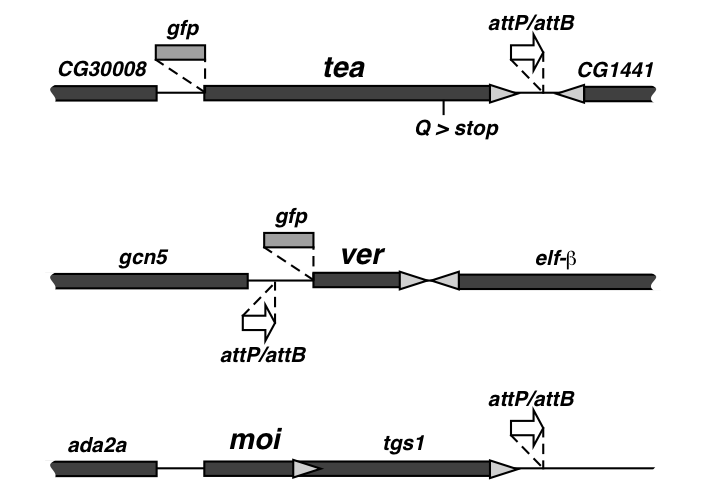

Supplement: S1 Fig — (TIFF) [file pgen.1006435.s002.tiff]

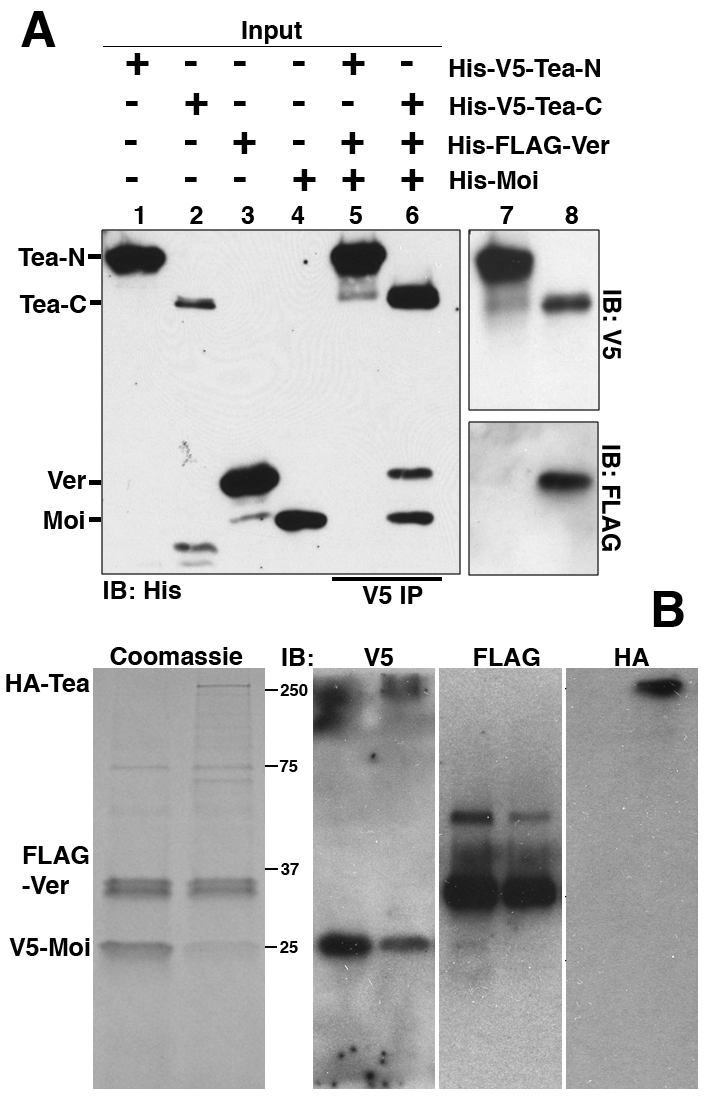

Supplement: S2 Fig — (TIF) [file pgen.1006435.s003.tif]
