# Supplementary material for: MTV, an ssDNA Protecting Complex Essential for Transposon-Based Telomere Maintenance in Drosophila
Source: PLoS Genet. 2016 Nov 11;12(11):e1006435. doi: 10.1371/journal.pgen.1006435 (PMC5105952; doi:10.1371/journal.pgen.1006435)
Supplement: S1 Table — (PDF) [file pgen.1006435.s004.pdf]

primer names	primer sequences
BTM-EcoRI-Ver-up	TAGAATTC**ATGGATTTTAATCAGAGTTTC**
BTM-Ver-SalI-down	**AC**GTCGACCTA**TTTATTTGTTGTATTCTGCATTG**
BTM-EcoRI-Moi-up	TAGAATTC**ATGTCCCTGGTGCCAGAAGCC**
BTM-Moi-SalI-stop	ACGTCGACGcta**TTTCTCGATCAGACTTCTCATC**
pACT-NcoI-Ver-N-F	cgccatggATGGATTTCAATCAGAGTTTC
pACT-EcoRI-Ver-down	TCGAATTCCTA**TTTATTTGTTGTATTCTGCATTG**
pACT-NcoI-Moi-F	taccatggATGTCCCTGGTGCCAGAAGCC
pACT-EcoRI-Moi-down	TCGAATTCCTA**TTTCTCGATCAGACTTCTCATC**
pACT-XmaI-Tea-F-1	tacccgggttATGTATCCAGTGTCCTTTGCG
pACT-XhoI-Tea-R-619	cgctcgagctaCTTAGGATTGGGGCAATTGAA
pACT-XmaI-Tea-F-620	agcccgggttATGCTGGCTAAGCTAAAACAA
pACT-XhoI-Tea-R-1587	CGCTCGAGCTATTTAGGAATCACAGAAACCTG
pACT-XmaI-Tea-F-1588	tacccgggttAAGAGACAGGCAGCCAGCCCAC
pACT-XhoI-Tea-R-1878	cgctcgagctaTTAAGTAACCAATTTACATTT
pACT-NcoI-N-Moi-F	taccatggATGTCCCTGGTGCCAGAAGCC
pACT-EcoRI-N-Moi-R	tcgaattcctaAATGCAGTAGCGGCCGGTAC
pACT-NcoI-C-Moi-F	taccatggGTGCGTGGTGAAGTGGTGCTAC
pACT-EcoRI-Moi-down	TCGAATTCCTA**TTTCTCGATCAGACTTCTCATC**
pACT-NcoI-Ver-N-F	cgccatggATGGATTTCAATCAGAGTTTC
pACT-EcoRI-Ver-N-R	tagaattcctaCGGATGCACGTCGTCTGTGATG
pACT-BamHI-Ver-68	ctggatccATTCACTTCTGCACCTGCATC
pACT-EcoRI-Ver-down	TCGAATTCCTA**TTTATTTGTTGTATTCTGCATTG**
pBTM-CmnoLex-F	atttcaagctataccaagcatacaatcaactccaagcttgaattaattccgGGCGCGCC**AGCCAGTATACACTCCGCTA**
pBTM-Cm-Moi-R	aagtagcagtacttgggcgccgaggtagaggcttctggcaccagggacatGGCGCGCC**CTGTGGAACACCTACATCTG**
pBTM-Cm-Ver-R	aagttatccagctggctttctatgtcctcgaaactctgattaaaatccatGGCGCGCC**CTGTGGAACACCTACATCTG**
pBTM-CmKm6-F	tgcacaaacaatacttaaataaatactactcagtaataacctatttcttagcGGCGCGCC**AGCCAGTATACACTCCGCTA**
PBTM-CmKm6-R	gcattggtgactattgagcacgtgagtatacgtgattaagctcacaaaggc**ATCATCGATGAATTCGAGCTCG**
QE-SphI-Moi-up	TAGCATGC**ATGTCCCTGGTGCCAGAAGCC**
QE-Moi-HindIII-down	CGAAGC**TTTCTCGATCAGACTTCTCATC**
pET-NdeI-Verflag	tgCATATGgactacaaagacgatgacgacaagGATTTCAATCAGAGTTTCG
pET-EcoR-Ver-R	**AC**GaattCCTA**TTTATTTGTTGTATTCTGCATTG**
pET-NdeI-1f	tgCATATGGGTAAGCCTATCCCTAACCCTCTCCTCGGTCTCGATTCTACGCCCGGGtatccagtgtcctttgcggc
pET-XhoI-348r	agCTCGAGTTAgggcctgcgcgaatcaagtcc
pET-NdeI-1584f	tgCATATGGGTAAGCCTATCCCTAACCCTCTCCTCGGTCTCGATTCTACG*GGATCC*caggcagccagcccacgtttg
pET-XhoI-1876r	agCTCGAGttaagtaaccaatttacattt
V5-AscI+Cm	GGTAAGCCTATCCCTAACCCTCTCCTCGGTCTCGATTCTACGggcgcgcc**AGCCAGTATACACTCCGCTA**
AscI+Cm	ggcgcgcc**CTGTGGAACACCTACATCTG**
attL1-Ver	CAAATAATGATTTTATTTTGACTGATAGTGACCTGTTCGTTGCAACAAATTGATGAGCAATGCTTTTTTATAATGCCAACTTTGTACAAAAAAGCAGGCTccATGGATTTCAATCAGAGTTTC
attL2-Ver	*CAAATAATGATTTTATTTTGACTGATAGTGACCTGTTCGTTGCAACAAATTGATAAGCAATGCTTTCTTATAATGCCAACTTTGTACAAGAAAGCTGGGTc***CTATTTATTTGTTGTATTCTGCATTG**
attL1-Moi	*CAAATAATGATTTTATTTTGACTGATAGTGACCTGTTCGTTGCAACAAATTGATGAGCAATGCTTTTTTATAATGCCAACTTTGTACAAAAAAGCAGGCT*cc**ATGTCCCTGGTGCCAGAAGCC**
attL2-Moi	*CAAATAATGATTTTATTTTGACTGATAGTGACCTGTTCGTTGCAACAAATTGATAAGCAATGCTTTCTTATAATGCCAACTTTGTACAAGAAAGCTGGGT*c**CTATTTCTCGATCAGACTTCTCATC**
EcoRI-3xFlag-Ver-up	TAGAATTC**atgtcgtactaccatcaccatcaccatcacgattacgatatcGATTTTAATCAGAGTTTC**
Not-Ver-R	atGCGGCCGCctatttatttgttgtattctgcattg
EcoRI-V5-Moi-up	TAGAATTC**atgtcgtactaccatcaccatcaccatcacgattacgatatcTCCCTGGTGCCAGAAGCC**
Not-Moi-R	atGCGGCCGCctatttctcgatcagacttctcatc
NotI-3xHA-TEA-f	atGCGGCCGCtCTGGACTGGAAGTTCTGTTCCAGGGGCCCCACGGATCCGGCGGCGGTGGTtatccagtgtcctttgcggc
XhoI-Tea-R-1878	cgctcgagctaTTAAGTAACCAATTTACATTT
